# Perception of epidemic's related anxiety in the General French Population: a cross-sectional study in the Rhône-Alpes region

**DOI:** 10.1186/1471-2458-10-191

**Published:** 2010-04-13

**Authors:** Mitra Saadatian-Elahi, Françoise Facy, Corinne Del Signore, Philippe Vanhems

**Affiliations:** 1 Hospices Civils de Lyon, Hôpital Edouard Herriot, Service d'Hygiène, Epidémiologie et Prévention, 5 place d'Arsonval, Lyon F-69437, France; 2 Université de Lyon; Université Lyon 1; CNRS, UMR 5558, Laboratoire de Biométrie et Biologie Evolutive, Equipe Epidémiologie et Santé Publique, 8 avenue Rockefeller, Lyon F-69373, France; 3 Université Claude Bernard, Lyon 1, INSERM Unité 28 Epidémiologie des conduites addictives Le Vésinet et UMRETTE, France

## Abstract

**Background:**

To efficiently plan appropriate public health interventions during possible epidemics, governments must take into consideration the following factors about the general population: their knowledge of epidemics, their fears of and psychological responses to them, their level of compliance with government measures and their communities' trusted sources of information. However, such surveys among the French general population are rare.

**Methods:**

A cross-sectional study was conducted in 2006 in a representative sample of 600 subjects living in the Rhône-Alpes region (south-east France) to investigate self-reported knowledge about infectious diseases and anxiety generated by epidemic risk with particular reference to avian influenza. Data on reactions to potentially new epidemics and the confidence level in various sources of information were also collected.

**Results:**

Respondents were most knowledgeable about AIDS, followed by avian influenza. Overall, 75% of respondents had adequate knowledge of avian influenza. The percentage was even higher (88%) among inhabitants of the Ain district, where an avian influenza epidemic had previously been reported. However, 39% expressed anxiety about this disease. In total, 20% of respondents with knowledge about avian influenza stated that they had changed their behaviours during the epizooty. Epidemics were perceived as a real threat by 27% of respondents. In the event of a highly contagious outbreak, the majority of respondents said they would follow the advice given by authorities. The study population expressed a high level of confidence in physicians and scientists, but had strong reservations about politicians, deputies and the media.

**Conclusions:**

Although the survey was conducted only four months after the avian influenza outbreak, epidemics were not perceived as a major threat by the study population. The results showed that in the event of a highly infectious disease, the population would comply with advice given by public authorities.

## Background

Anxiety generated by epidemics in the general population is poorly understood. A deeper understanding of this phenomenon would allow us to better anticipate and control potentially inappropriate and/or unexpected behaviours in the event of a massive epidemic like influenza. People's feeling of risk is a subjective judgment. Each individual has unique characteristics resulting in unique behaviour towards any specific risk. This behaviour springs from a combination of factors such as personal values, social and cultural background, gender and education [1,2]. The population's knowledge level about the disease plays also an important role in response to an epidemic crisis and could impact on collective attitudes [3,4]. The media also play a role in influencing public perceptions of epidemics or pandemics as they tend to bias perceptions by under- or over-estimating risk factors, morbidity and mortality [5].

Public health interventions have been planned to control future influenza pandemics, but these national measures were proposed by health professionals who had no knowledge of risk perception in the population. In France, only two studies have investigated the perception of disease-related risk in the general population [6,7], but they did not include infectious diseases.

The objective of this cross-sectional study was to explore the perceived anxiety of epidemic risk in the general population of the Rhône-Alpes region, with particular reference to avian influenza. Located in south-east France and bordering Italy as well as Switzerland, the Rhône-Alpes region has more than six million inhabitants.

## Methods

This survey was conducted by computer-assisted telephonic interview between June 27 and July 5, 2006 by IPSOS, a professional survey institute specialized in surveys in the general French population. The survey was undertaken in the Rhône-Alpes region, which includes eight districts (Ain, Ardèche, Drôme, Isère, Loire, Rhône, Savoie, and Haute Savoie). Individuals aged 18 years and over were eligible to participate. Respondents were selected by the non-probabilistic "quota" method, which consists of questioning a sample of people with the same socio-demographic characteristics as the whole population. The quotas to be respected were based on data provided by the last INSEE (National Institute of Statistics and Economic Studies) census, according to gender, age and profession, and stratified by area of residence.

Of the 800 people contacted by telephone, 600 agreed to answer the questionnaire, giving a 75% response rate. The study was carried out four months after an epizooty of avian influenza in the Ain area, a district with more than 500,000 inhabitants.

Data were collected by a specially-designed questionnaire. The first part of the questionnaire included basic demographic questions about age, gender, education level, marital and employment status. The two following questions focused on self-evaluated knowledge, based on a scale from 1 (don't know at all) to 5 (know very well), and the extent of anxiety based on a scale from 1 (not at all anxious) to 4 (very anxious), about severe acute respiratory syndrome (SARS), acquired immunodeficiency syndrome (AIDS), viral hepatitis, nosocomial infections, meningitis and avian influenza. Subjects who had previously indicated self-reported knowledge of avian influenza were invited to answer questions about the disease's transmission routes, their behaviours at the time the disease had occurred in France, and their opinion on preventive measures taken by the French health authorities, also based on a scale from 1 (very confident) to 4 (not at all confident). This section was followed by questions on reactions to the likelihood of a new epidemic, sources of information, trust in these sources and level of confidence in public health authorities. In order to determine the importance of epidemics among other preoccupations, the questionnaire concluded by asking the respondents to classify their three main threats for the French society among the followed items: cancers, pollution, unemployment, terrorism, obesity and epidemics.

The study was submitted to and approved by the French Data Protection Agency (CNIL).

The data were described by appropriate statistics for categorical or continuous variables. The analysis was carried out with SPSS version 11.0 (SPSS Inc., Chicago, IL).

## Results

Respondents (47% male) were classified into five age categories: 18-24, 25-34, 35-44, 45-59, 60 years and above. Overall, 63% were married, and 42% had at least 1 child. In total, 19% had no educational qualifications, 51% had high school diplomas, and 29% had university degrees (Table 1).

**Table 1:** Baseline characteristics of the study population: June-July 2006, Lyon-France (n = 601).

Gender (%)	
	
Men	47
Women	53
**Age (years)**	
	
18-24	11
25-34	18
35-44	22
45-59	25
> 60	24

**District (%)**	
	
Ain	9
Ardèche	5
Drôme	8
Isère	20
Loire	12
Rhône	27
Savoie	7
Haute-Savoie	12

**Profession of household head**	
	
Farmers, craftsmen, tradesmen,	8
Professionals and self-employed	12
Service industry workers	15
Employees	11
Manual workers	25
Retired	23
Other unemployed persons	6

Table 2 summarizes the respondents' self-evaluated knowledge of infectious diseases and their level of anxiety. AIDS was the most well-known infectious disease since 93% of respondents confirmed they had adequate knowledge of it. Avian influenza was second (75%). The percentage of subjects with adequate knowledge of avian influenza was even higher among those who worked with animals (78%), had children (79%) and lived in the Ain district (88%), an area in France where avian influenza epidemic had previously been reported [8] (data not shown). Meningitis came in third (68%), followed by viral hepatitis (56%) and nosocomial infections (48%). In total, 40% of respondents indicated they had no knowledge of SARS.

**Table 2:** Self-reported knowledge of infectious diseases and perceived personal risk: June-July 2006, Lyon-France.

Self-evaluated level of knowledge (n = 601)					
	**%**	**%**	**%**	**%**	**%**

	**Not at all**	**Low**	**Medium**	**Well**	**Very well**

AIDS	1	1	5	46	47
SARS	42	14	20	16	6
Avian Influenza	6	6	13	48	27
Viral hepatitis	7	9	27	45	12
Nosocomial infections	17	12	22	33	15
Meningitis	2	6	24	47	21

**Level of anxiety among subjects with knowledge of the diseases**					

	**%**	**%**	**%**	**%**	

	**Not at all anxious**	**Somewhat anxious**	**Anxious**	**Very anxious**	

AIDS (n = 595)	8	24	50	13	
SARS (n = 345)	16	34	34	8	
Avian Influenza (n = 567)	26	35	30	9	
Viral hepatitis (n = 558)	11	38	39	8	
Nosocomial infections (n = 500)	8	24	50	13	
Meningitis (n = 589)	7	27	42	18	

With regard to the anxiety generated by the above-mentioned diseases, 63% expressed concern about nosocomial infections, 61% about AIDS, 59% about meningitis and 46% about viral hepatitis. The reported anxiety of meningitis was significantly higher among subjects who had children (66%). The extent of anxiety of SARS and avian flu was in general lower, with 42% and 39%, respectively, expressing concern about these diseases. Analysis by gender indicated that, except for nosocomial infections, women were significantly more worried than men (data not included). Over 60% of women reported feeling anxious about all the infectious diseases mentioned, compared to 35% of men on average. A higher level of anxiety for nosocomial infections and SARS was associated with lower socio-economic status (68% vs 63% on average and 48% versus 42% on average respectively).

Table 3 enumerates the self-reported knowledge of infectious diseases by gender, age categories and socio-economic status as well as items considered to be a major threat for the study population. Women were generally better informed than men. Knowledge about nosocomial infections was significantly higher among older subjects (>60 years old), compared to other age categories, while 18- to 34-year-olds were significantly more familiar with AIDS. Knowledge of meningitis was significantly higher among 35- to 44-year-olds. Respondents belonging to lower socio-economic classes had significantly less knowledge of nosocomial infections and viral hepatitis. Cancers were reported as the most important threat for the study population (74%) followed by diseases related to atmospheric pollution (62%), unemployment (50%) and terrorism (46%). Epidemics were perceived as a real risk by 27% of respondents.

**Table 3:** Self- reported knowledge of infectious diseases according to gender, age and socio-economic status and principal fears of French society, as perceived by the study population: June-July 2006, Lyon-France.

Percentage of subjects with knowledge about infectious diseases (n = 601)						
	**AIDS**	**Avian influenza**	**Meningitis**	**Viral hepatitis**	**Nosocomial** **infections**	**SARS**

Total (%)	93	75	68	56	48	23
						
Men (%)	92	74	64	47*	45	22
Women (%)	93	76	71	65*	51	23
						
**Age categories (years)**						
18-24	99	74	63	35*	32*	19
25-34	93	77	66	51	39*	20
35-44	96	81	77*	62	50	24
45-59	92	71	67	62	51	20
60 and over	88*	73	65	59	59*	27
						
**Socio-economic status**						
High	95	76	72	63	55	25
Medium	94	81	68	60	54	21
Low	92	72	67	53*	44*	22
						
First evoked (%)	38	21	14	15	8	4
Second evoked (%)	36	41	36	31	28	23
Total (%)	74	62	50	46	36	27

**Fears** **(n = 601)**						

	**Cancer**	**Pollution**	**unemployment**	**Terrorism**	**Obesity**	**Epidemics**

First evoked (%)	38	21	14	15	8	4
Second evoked (%)	36	41	36	31	28	23
Total (%)	74	62	50	46	36	27

*: significantly different to the total (p < 0.05)

AIDS: Acquired immune deficiency syndrome

SARS: Severe Acute Respiratory Syndrome

All subjects who indicated they had knowledge of avian influenza (n = 567, 88%) were invited to answer questions about its routes of transmission, their individual behaviours at the time of the outbreak in certain poultry farms in France, and their opinions about measures taken by French health authorities (Table 4). Contact with contaminated poultry was the main transmission route (n = 459, 81%) cited. Most respondents (n = 430, 76%) did not consider poultry or egg consumption a route of transmission, but their opinions about possible transmission by close contact with contaminated individuals were more equivocal. Overall, 36% (41% of males) believed that avian influenza could undergo inter-human transmission, while 54% said the opposite (65% in high socio-economic categories). Overall, behavioural changes were more significant among women. Only a small percentage indicated that they had stopped eating poultry (n = 113, 20%) or avoided travelling to countries at risk (n = 107, 19%) or to places such as farms where the risk of contamination was high (n = 96, 17%). The percentage of subjects who avoided travelling was greater among those aged 45 years and over (24%) and among women (22%). An even smaller percentage (n = 28, 5%) indicated they had consulted a physician or bought antiviral drugs (n = 23, 4%). The percentage was higher (7%) among the elderly (>60 years old) and subjects belonging to lower socio-economic categories (9%). The behavioural changes were still in effect at the time of the survey but in a smaller proportion, suggesting that the media had influenced the population's attitude.

**Table 4:** Avian influenza: Reported transmission pattern and behaviour during the 2005 epizooty and at the time of the survey: June-July 2006, Lyon-France.

Reported transmission route (n = 567)					
	**Yes** **(%)**	**No** **(%)**	**Don't know** **(%)**		
	
Contacts with contaminated poultry	81	17	3		
Consumption of eggs or poultry	21	76	3		
Contacts with contaminated subjects	37	49	15		
Intra-human transmission	36	54	10		

**Behaviours** **(n = 567)**					

	**In 2005**			**At the time of survey**	
	
	**Yes** **(%)**	**No** **(%)**		**Yes** **(%)**	**No** **(%)**
Do not eat poultry	20	80		10	90
Avoid or cancel travel to countries at risk	19	81		13	87
Avoid public places or at risk persons	17	83		6	94
Consult physicians for more information	5	94		2	98
Buy antiviral drugs	4	96		2	98
Others	3	96		1	99

**Perception of measures taken by the French health authorities**					

	**Yes** **(%)**	**No** **(%)**	**Don't know** **(%)**		
	
Know the measures (n = 567)	82	18		-	
Express confidence in the measures (n = 466)	68	32		-	
Think measures are adapted to the situation (n = 466)	68	32		-	

Of the 82% (n = 465) who indicated they were aware of the decisions taken by the French authorities during the avian influenza epizootic, 68% approved them. The degree of approval was even higher (79%) among subjects over 60 years of age. Moreover, 76% of respondents expressed their solidarity with the professions involved, such as poultry stockbreeders.

When asked about their possible behaviours during official announcement of a highly contagious disease in France, almost all respondents said they would follow the advice of their physician (96%) or the public authorities (92%) (Table 5). The rate rose to 97% in higher socio-economic categories. Approximately 80% said they would buy existing drugs. Fewer than 50% indicated that they would avoid contact with others (30%) or leave the country (12%). Most subjects stated that they would follow the preventive measures announced by public authorities. As a result, 96% would accept being quarantined, 91% would wear a mask in public places, and 97% would wash their hands more than 10 times a day. A large percentage (83%) would also encourage their friends and family to comply with the official recommendations. However, only 21% (27% among 45- to 59-year-olds) would report those who did not follow preventive measures.

**Table 5:** Behaviours in the event of official announcement of a highly contagious disease in France: June-July 2006, Lyon-France (n = 601).

Individual behaviours			
	**Yes** **(%)**	**No** **(%)**	**Don't know** **(%)**
	
Follow advice given by the family physician	96	4	0
Follow advice given by public health authorities	92*	6	2
Buy existing medications	80	14	6
Avoid public places (restaurants, stadiums, theatres)	47	48	5
Stay home	30*	64	6
Avoid hospitals	44	49	7
Leave France	12*	85	3

**Follow prevention measures**			

	**Yes** **(%)**	**No** **(%)**	**Don't know** **(%)**
	
Accept to be quarantined	96§	3	1
Wear a mask in public areas	91*	7	2
Wash hands 10 times a day with disinfectants	97*#	3	1

**Compliance with preventive measures**			

	**Yes** **(%)**	**No** **(%)**	**Don't know** **(%)**
	
Would personally comply	91§	5	4
Would make children comply (n = 252)	98#	1	2
Would encourage friends and family to comply with official recommendations	83	14	3
Would report individuals who do not follow official recommendations	21	75	4

* Significantly greater among those belonging to high socio-economic status groups (p < 0.05)

§Significantly greater among those belonging to medium socio-economic status groups (p < 0.05)

# Significantly greater among women (p < 0.05)

**Figure 1:**
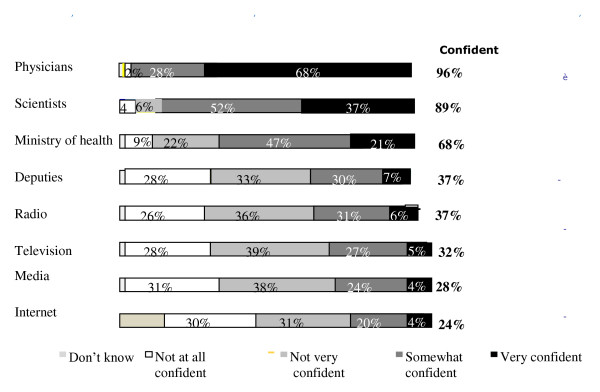
Level of trust of the study population in different sources of information: June-July 2006, Lyon-France.

In terms of trusted sources of information in case of occurrence of a highly contagious disease, respondents were mostly confident in their physicians (97%) and scientists (89%), followed by the Ministry of Health (68%). The study population had strong reservations about politicians, deputies and the media, and under 10% said they would not fully trust them (Figure 1).

## Discussion

Few population-based studies have been carried out in France to investigate the level of anxiety generated by epidemic risk in the general population. The main objectives of this cross-sectional survey were to evaluate: i) extent of anxiety of epidemic risk; ii) knowledge level about infectious diseases; iii) reactions in the event of an epidemic or pandemic; and iv) confidence level in various sources of information.

Recruitment by the quota method enabled the selection of a representative sample, but the sample size was relatively small and limited to one region. Larger national studies are warranted to gain a broader understanding of the knowledge and anxiety pertaining to epidemic risk in the French population.

The study was carried out four months after the avian influenza epizootic occurred in France, providing the opportunity to evaluate the short- and medium-term impact of an epidemic on individual behaviours. About one quarter of the study population was concerned by epidemics, probably because of the public's overall confidence in the healthcare system and in its ability to handle such situations. Indeed, as soon as suspicion of avian influenza in a poultry breeding, surveillance measures including confiscation of the breeding, and controls of all movements (people, other animals, etc) in the suspected area were set up to limit the propagation of the infection. Relative powerlessness in the face of such a large event could also be a contributing factor. Similar arguments have been reported in a qualitative survey conducted in the U.K. and the Netherlands [9]. Moreover, the majority of human epidemics reported by media have occurred in foreign countries (avian influenza and SARS in Asia, cholera, and hemorrhagic fever in Africa). Severe cases of infections such as the outbreak of legionnaire's disease reported by the media in France are rather sporadic. Pursuant to the present study, an outbreak of *Clostridium difficile *type 027 occurred in Northern France [10]. However, to our knowledge, no data on worries and concerns of the general population have been published for this disease.

The higher reported concerns for cancers, unemployment and meningitis, reported also by other French studies [7] may be due to their higher prevalence and strong mediatisation in France.

In February 2006, the highly pathogenic avian influenza (HPAI) H5N1 virus was isolated in the Ain district, an important migration and wintering waterfowl area of the Rhône-Alpes region. The epizootic lasted two months and may explain why subjects living in this area were well informed about the disease. France was the first European Union country where non-wild birds had been implicated. After the epizootic, extensive media attention led to a decrease in visits to this area, particularly to a public bird park located in the district. These results emphasize the importance of implementing effective communication strategies for the general public. In the early phase of an outbreak, communication is usually targeted at a segment of the population considered at risk. In the rest of the population, this could create a feeling of complacency and lead to stigmatization and discrimination against the population labelled to be potentially at risk. Such attitudes have been reported during SARS epidemics [9,11-14]. Another striking example is provided by AIDS. The earliest education and prevention campaigns were mainly directed at men who have sex with men and at young people. This approach may have contributed to an increase in the incidence of AIDS among heterosexuals and higher age categories [15-19].

Less than 20% of respondents with adequate knowledge of avian influenza reported avoiding travel to at-risk countries, but the percentage was higher among older subjects. Similar results have been obtained in a recent survey conducted among Finnish tourists in Asia during the avian influenza outbreak [20].

With the exception of SARS, the majority of respondents believed they had adequate information about other infectious diseases. The lower level of knowledge about SARS in the study population may be related to the absence of cases in France. The mean knowledge score of SARS was 1.2 in Europe and 1.7 in Asia (p < 0.0001) in an international survey involving over 3,000 respondents in five European and three Asian countries [21]. The knowledge level about nosocomial infections and AIDS was substantially different between age categories. Younger people felt they were more at risk of acquiring a sexually-transmitted disease than they were of an infection during hospitalization and were, therefore, more likely to be informed about the former. They may associate hospital infections with old age and, for this reason, may be less concerned about nosocomial infections.

A civic-minded attitude emerged from answers under the scenario of the occurrence of a highly infectious disease. The population said it would follow advice given by public authorities and would agree to be quarantined, to wash their hands several times a day and wear a face mask. However, we should exercise caution in extrapolating the observed feeling of relative complacency towards epidemics in real situations. During serious outbreaks of disease, the population would probably move from a phase of calm and serenity to a phase of major panic without going through an intermediate stage. The SARS epidemic was an excellent example of this situation [22]. Compliance with advice could also reflect anxiety and psychological distress [23]. A positive dose-response gradient between self-protection and level of anxiety has been reported by a longitudinal telephone survey in Hong Kong [24]. It is noteworthy that panic caused by local outbreaks may lead to worldwide reactions because the spread of pathogenic agents in modern societies is facilitated by extensive air travel.

Effective public health action is feasible by taking into consideration the public's knowledge of epidemics, their fears, psychological responses and compliance level with public health measures. International collaboration aimed at improving the understanding of these factors would alleviate public apprehension and enhance preparedness for and control of epidemic crises.

The results of the current survey showed that a large majority of respondents would not trust politicians and the media but would follow the advices of their physicians. Native population of two other European countries reported also most confidence in their physicians while foreign media and family/friends were the most trusted sources of information among Asian communities living in these countries [25]. Identifying the communities' trusted sources of information would help authorities to effectively present preventive and educational messages. Governments should proactively reassure the population by becoming more involved in this public health issue. Suitable communications would reassure the population and improve control of the epidemic situation.

Some limitations of the study should be addressed. Because of the use of a phone survey, the questionnaire had a limited number of items. Content questions to validate the actual self-reported knowledge of infectious diseases were primarily focussed on avian influenza. However, with the exception of SARS, other cited infectious diseases were well-known prevalent and/or mediatised diseases. The results of this exploratory study could be the basis for more elaborated and comprehensive studies on risk perception in the French population.

The recent H1N1 flu pandemic alert [26] is a real time situation in which the public's risk perception could be explored. Population-based surveys become critical in the current context of the recent transmission of swine influenza viruses to humans. Unlike avian influenza, inter-human transmission of this pathogenic virus has been established [26]. Furthermore, it has spread quickly across countries and borders, with several countries reporting laboratory- confirmed cases of the disease. Since the first case was identified in Mexico in early April 2009, various national and international health and governmental bodies have made plans to monitor and control this new outbreak. However, to better coordinate such plans, they must acquire information about the population's knowledge level of the disease, their feelings about it, the acceptability of a possible vaccine program, etc.

## Conclusions

Despite the occurrence of new epidemics such as SARS and avian influenza just before this study was conducted, epidemics were not perceived as the main concern of the study population. Avian influenza was the second well known infectious disease after AIDS. The results showed that in the event of an epidemic, the population would follow the advice given by public authorities and would also convince their entourage to meet the recommendations.

## Competing interests

The authors declare that they have no competing interests.

## Authors' contributions

PV conceived the study. PV and MSE designed the study and drafted the manuscript. FF participated in questionnaire design and helped to draft the manuscript. CDS assisted in drafting the manuscript. All authors read and approved the final manuscript.

## Pre-publication history

The pre-publication history for this paper can be accessed here:

http://www.biomedcentral.com/1471-2458/10/191/prepub
